# Advances in decomposing complex metabolite mixtures using substructure- and network-based computational metabolomics approaches

**DOI:** 10.1039/d1np00023c

**Published:** 2021-06-18

**Authors:** Mehdi A. Beniddir, Kyo Bin Kang, Grégory Genta-Jouve, Florian Huber, Simon Rogers, Justin J. J. van der Hooft

**Affiliations:** Université Paris-Saclay, CNRS, BioCIS 5 rue J.-B Clément 92290 Châtenay-Malabry France; Research Institute of Pharmaceutical Sciences, College of Pharmacy, Sookmyung Women's University Seoul 04310 Republic of Korea; Laboratoire de Chimie-Toxicologie Analytique et Cellulaire (C-TAC), UMR CNRS 8038, CiTCoM, Université de Paris 4, Avenue de l’Observatoire 75006 Paris France; Laboratoire Ecologie, Evolution, Interactions des Systèmes Amazoniens (LEEISA), USR 3456, Université De Guyane, CNRS Guyane 275 Route de Montabo 97334 Cayenne French Guiana France; Netherlands eScience Center 1098 XG Amsterdam The Netherlands; School of Computing Science, University of Glasgow Glasgow G12 8QQ UK; Bioinformatics Group, Wageningen University 6708 PB Wageningen The Netherlands justin.vanderhooft@wur.nl

## Abstract

Covering: up to the end of 2020

Recently introduced computational metabolome mining tools have started to positively impact the chemical and biological interpretation of untargeted metabolomics analyses. We believe that these current advances make it possible to start decomposing complex metabolite mixtures into substructure and chemical class information, thereby supporting pivotal tasks in metabolomics analysis including metabolite annotation, the comparison of metabolic profiles, and network analyses. In this review, we highlight and explain key tools and emerging strategies covering 2015 up to the end of 2020. The majority of these tools aim at processing and analyzing liquid chromatography coupled to mass spectrometry fragmentation data. We start with defining what substructures are, how they relate to molecular fingerprints, and how recognizing them helps to decompose complex mixtures. We continue with chemical classes that are based on the presence or absence of particular molecular scaffolds and/or functional groups and are thus intrinsically related to substructures. We discuss novel tools to mine substructures, annotate chemical compound classes, and create mass spectral networks from metabolomics data and demonstrate them using two case studies. We also review and speculate about the opportunities that NMR spectroscopy-based metabolome mining of complex metabolite mixtures offers to discover substructures and chemical classes. Finally, we will describe the main benefits and limitations of the current tools and strategies that rely on them, and our vision on how this exciting field can develop toward repository-scale-sized metabolomics analyses. Complementary sources of structural information from genomics analyses and well-curated taxonomic records are also discussed. Many research fields such as natural products discovery, pharmacokinetic and drug metabolism studies, and environmental metabolomics increasingly rely on untargeted metabolomics to gain biochemical and biological insights. The here described technical advances will benefit all those metabolomics disciplines by transforming spectral data into knowledge that can answer biological questions.

## Abstract 2

This review highlights the key computational tools and emerging strategies for metabolite annotation, and discusses how these advances will enable integrated large-scale analysis to accelerate natural product discovery.

## Graphical abstract

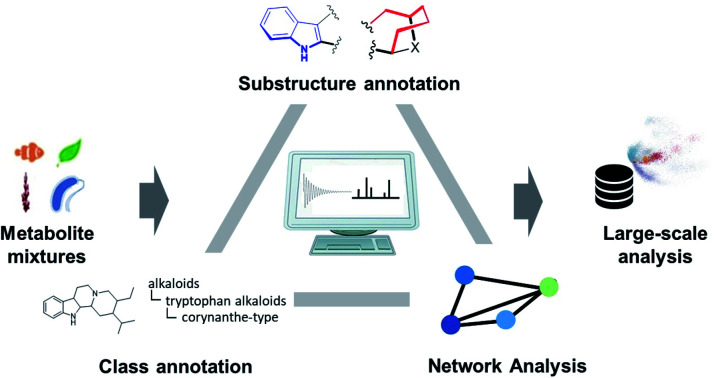

## 1 Introduction

Complex metabolite mixtures are found everywhere in and around us. Whether you study plant or microbial extracts, environmental samples, or human urine or plasma, these samples include vast numbers of chemically diverse molecules whose structures are mostly unknown up to date.^1,2^ However, such molecules can play important physiological, biochemical, ecological, or diagnostic roles: in plants and microbes, they can serve as messengers or as antibacterial or antifungal agents, whereas in human biofluids molecules can be signaling molecules, biomarkers of disease, or markers of food intake or microbial activity. In plants and microbes, we typically refer to such molecules as natural products, or specialized metabolites.^3^ Specialized metabolites were previously called secondary metabolites, because they were thought not to be directly involved with primary functions such as growth, reproduction, or development, and they were assumed to be useless waste products in early days. Instead, now they are known to have advantageous effects on their producers in various “indirect” ways, for example, by repelling herbivores thus preventing them from eating the plant. Biosynthetic pathways for specialized metabolites show great diversity at the level of taxa, organs, and tissues;^4–6^ opposed to the central metabolism which is highly conserved. Due to this reason, they are now known as specialized metabolites.

Knowing the structures and roles of all molecules in complex mixtures would greatly enhance our knowledge of the ecological function of plants and microbes in the ecosystem. To better tackle the chemical complexity of plants and microbes, analytical and computational approaches have been developed over the last decade or two.^7,8^ More recently, the first studies have started to comprehensively analyze the specialized metabolome through integrated analysis of tens to a few hundreds of samples.^9–11^

Metabolomics is the field of study that aims to get a comprehensive view of the molecular contents of organisms. Typical parts of a metabolomics study include sample collection, sample extraction, analytical measurement, data processing and analysis, and biochemical interpretation.^12^ Mass spectrometry (MS) and nuclear magnetic resonance (NMR) spectroscopy are the analytical workhorses of metabolomics. A major bottleneck in the metabolomics pipeline is metabolite annotation and identification,^13^*i.e.*, the assignment of structures to spectral data. This is a critical step in metabolomics workflows as the assigned structures are key to biochemical interpretation of the data. The classical route to characterize specialized metabolites from complex metabolite mixtures is through isolation and purification from the crude extract.^14^ First, the crude extract is separated into fractions using liquid chromatography (LC). Often, these fractions are further separated into individual components with more subtle LC approaches. The isolated and (semi-)purified molecules are measured with MS, sometimes supplemented by MS fragmentation (MS/MS), and extensive NMR measurements to collect sufficient spectral information to solve the puzzles of how many atoms of which sort there are, and how they are connected to each other. Thus, to come from the sample collection to a couple of known structures can take weeks or months since it is laborious work that involves analytical skills and chemical expertise. It has become clear that to do large-scale mining of the specialized metabolome, the classical reductive approach is not suitable. Therefore, increasingly, untargeted metabolomics approaches are employed that do a wide-screen survey of the chemical diversity in samples and generate information-dense high-resolution LC-MS/MS, or two dimensional (2D)-NMR profiles where interactions of neighboring protons or protons in close proximity are recorded.^14^ Similarly, such a 2D-NMR approach can be applied to protons and carbon atoms to study their interactions and learn structural features of the measured molecule. The two described analytical approaches both aim to accurately cover as much as molecules that are present in the complex mixtures, whereas also provide as much structural information as possible through mass fragmentation (MS/MS) spectra containing spectral patterns or NMR cross peaks indicative for atomic connections. To facilitate the data preprocessing, processing, analysis, and interpretation numerous computational metabolomics tools have been introduced.^7^ Whilst spectral databases containing reference spectra are growing for both MS and NMR, the matching rates for specialized metabolites to assign complete structures to spectral data remain low.^15^ Therefore, we here argue that substructure-based metabolomics workflows offer an interesting and feasible alternative since they target smaller parts of the molecules that are typically easier to structurally annotate. Most specialized metabolites in complex metabolite mixtures are not independent from each other: they can share common substructures or can be part of the same biosynthetic or biochemical pathway. Nature often reuses the same building blocks to create ever increasingly complex structures with diverse functions. In metabolomics data, such building blocks are expected to transpire into spectral patterns because groups of atoms that are in a similar constitution and chemical environment are likely to produce similar spectral signals. In other words, basic building blocks such as saccharides and specialized metabolite scaffolds are expected to produce the same or similar spectral signals even if they are present across different complete structures. It is this hypothesis that most of the currently available substructure discovery-based and chemical class-based metabolomics workflows use.

**Fig. 1:**
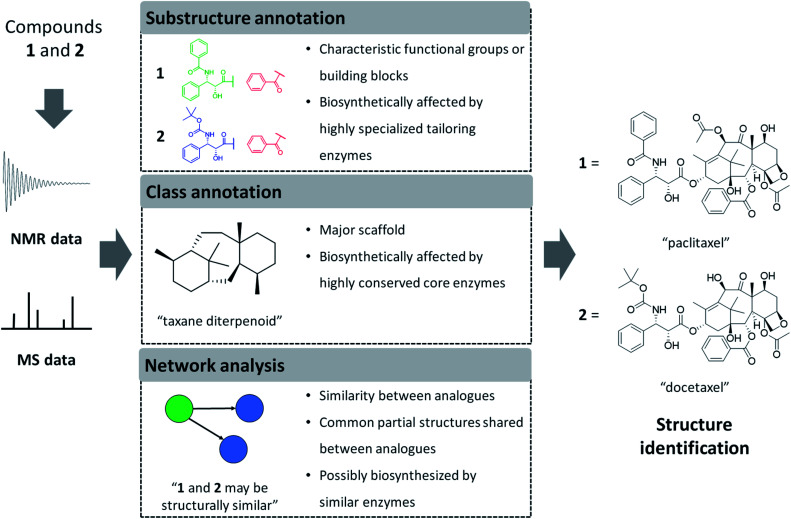
Computational interpretation of NMR or MS data provides complementary information related to chemical structures. Sometimes the entire structure can be identified directly, but more frequently we obtain knowledge on partial structures only. Here, we define three types of annotations and exemplify the information provided by each type of annotation by using an example of two structural analogues: paclitaxel and docetaxel. Please note that we define analogues as metabolites that share the majority of their structures with each other, in natural products-related research mainly because they are reactant pairs or because they are a group of biosynthetically or chemically related metabolites. Substructure annotations provide information on functional groups, building blocks, or scaffolds within a chemical structure. Class annotations give information on major backbone structures, which has been a major criterion for chemical ontology of natural products. Network analysis estimates chemical similarity between analogues through spectral similarity to form mass spectral networks. Structural annotations performed on spectral data of multiple metabolites can provide insights on the biosynthetic and chemical relationships between analytes as described in the figure. We do note that docetaxel is a semisynthetic compound designed based on the scaffold of paclitaxel.

In the following sections, we will highlight recently developed MS and NMR metabolite annotation tools that discover substructure patterns in metabolomics data and provide ways to annotate them and perform chemical compound class annotations. In this review, we define three different types of structural annotations: substructure annotation, class annotation, and network analysis (Fig. 1). Substructure annotation provides information on functional groups, building blocks, or scaffolds within a chemical structure, while chemical compound class annotation gives information on major backbone structures based on the biosynthetic origin or historical applications of the compounds – the latter has been a major criterion for chemical ontology of natural products. Network analysis does not provide structural information directly, but it reveals relationships between molecules, such as chemical similarity or shared substructures between metabolites, and it can enhance the annotation and structural characterization of multiple connected metabolites. We do note that the three types of annotation we recognize here are not completely independent of each other. For example, if a substructure annotation provides the structural information that coincides with the main scaffold, a chemical compound class annotation could be provided on the basis of it. Furthermore, the coexistence of substructure or chemical class features among multiple spectra could be a foundation of network analysis. As the chemical diversity of natural products is formed by the genetic diversity of biosynthetic genes, all of the annotations also provide biosynthesis-related information. Class annotation and network analysis can reveal congeners biosynthesized through a conserved upstream pathway, while substructure annotation could inform on the diverse tailoring reactions. To accentuate parts of the described workflows, we use case studies on monoterpene indole alkaloids and flavone glycosides. We will finish with our perspective on how substructure and network-based analyses will transform future metabolomics workflows to make them more scalable, more reliable, and allow for increased structural and functional interpretation of complex metabolite mixtures.

## 2 Substructure discovery-based MS-based metabolomics tools

### 2.1 Substructures as building blocks of metabolites

In natural extracts, multiple metabolites typically share the same or similar structural parts called substructures. The main reason for this is that the metabolic complexity found in nature is based on a finite number of molecular scaffolds that an organism can produce and that it typically decorates with various smaller (functional) groups. The instructions to construct, link, and decorate these building blocks are imprinted in the organism's genome. Hence, in complex metabolite mixtures, most metabolites are structurally somehow related to at least a few other metabolites through the use of shared or similar biosynthetic machinery. The recognition of the building blocks of metabolomics directly from spectral data is thus an attractive path to increase the annotation power of metabolomics workflows as they assist in assigning structures to key parts of metabolites and group them according to these annotations. Not only does this support metabolite annotation workflows that aim to solve complete metabolite structures; it does provide additional benefits: for some biological questions, solving the complete structures may not be necessary and using the structural information at the substructure level may be sufficient. For example, comparative metabolomics to link particular chemistry to a phenotype rather than metabolite structures could be done based on differential expression of substructure presence. The “old” way of spectral interpretation in MS/MS-based metabolomics (manual inspection) was actually this type of annotation; because the key data features of MS/MS, fragment ions and neutral losses, are fundamentally related to substructures. In this section, we will discuss recent machine learning-based tools that allow researchers to extract substructure information in the form of mass fragmentation patterns or molecular fingerprints from mass spectrometry fragmentation profiles. In some cases, this information is also used for annotations of complete structures.

### 2.2 Substructure discovery by MS2LDA

MS2LDA^16^ was developed for unsupervised substructure discovery through the extraction of recurring spectral patterns, termed Mass2Motifs, from mass spectrometry fragmentation (MS/MS) spectra. It is inspired by the natural language processing algorithm Latent Dirichlet Allocation (LDA),^17^ a method developed to decompose text documents into a series of topics, such as various categories for newspaper articles. This was a deviation from the more traditional document clustering techniques in that instead of attempting to place each document into a single topic it allowed documents to be made up of multiple topics. This resulted in the ability to decompose a set of text documents into a smaller set of more meaningful topics than would have been extracted with traditional document clustering.

As described in the previous section, metabolites can often be considered to be built up from discrete building blocks or substructures in much the same way that one can imagine documents being built up from topics. By representing MS/MS spectra in a bag-of-words formats (counts of the occurrence of different fragment and neutral loss features), MS2LDA applies an unsupervised LDA decomposition to MS/MS data resulting in Mass2Motifs (topics) made up of small numbers of co-occurring fragment and loss features. A single MS/MS spectrum can include multiple Mass2Motifs, with a probability score describing how much of the spectrum is made up of any particular Mass2Motif directly provided from the unsupervised LDA decomposition. An overlap score can also be computed that describes how much of any particular Mass2Motif occurs within a particular spectrum and, with the probability score, be used as a threshold to associate spectra to Mass2Motifs.^18^

**Fig. 2:**
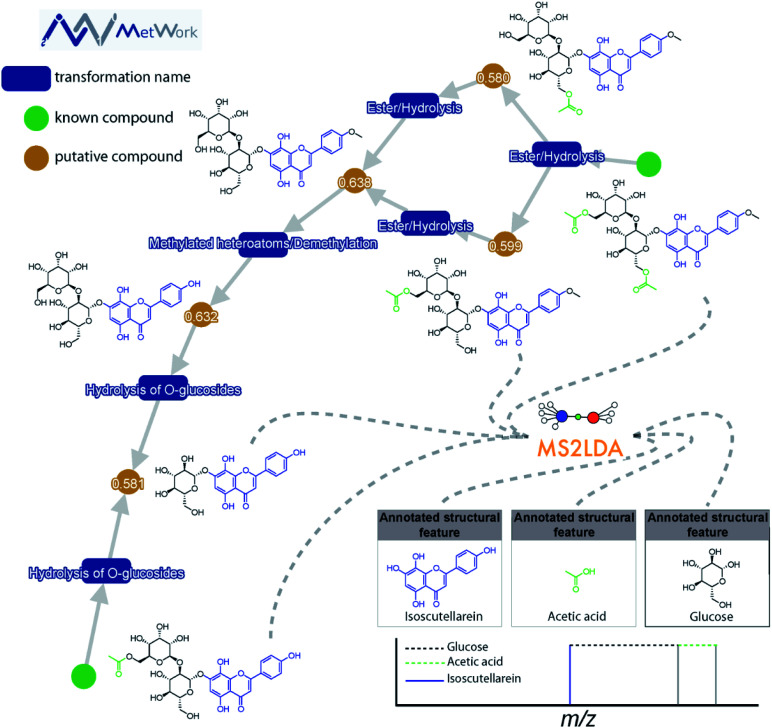
The structural annotation of plant flavone glycoside analogues by combining MS2LDA substructure finding and MetWork substance anticipation is highlighted. MetWork is a web application designed for specialized metabolites anticipation using *in silico* metabolism to predict metabolite structures, and it will be further highlighted in Section 4.2.3. The green nodes represent metabolites previously reported in literature to be present in the plant extract. In these two structures, several features are clearly identified using the Mass2Motif approach. As indicated in the bottom right corner, the main isoscutellarein motif could be assigned (mass fragment ion in blue). Two other features are highlighted, the acetic acid and the glucose (neutral losses). The identification of these motifs is in good agreement with the *in silico* metabolism products obtained using MetWork as can be seen in the displayed network. The cosine score between experimental and theoretical spectra of anticipated analogues are displayed on the orange nodes. Altogether, the combined approach enables the confident assignment of 5 additional acylated and/or glycosylated isoscutellarein analogues.

This decomposition can significantly aid the structural analysis of MS/MS data. Spectra can be grouped according to shared Mass2Motifs and prevalence of Mass2Motifs can be compared across different samples.^18^ Where many molecular spectra are hard to annotate directly based on library matching, *in silico* annotation tools, or prediction of mass spectra from structures, many Mass2Motifs can be annotated and these annotations can be added to any spectrum that includes that motif. For example, it was demonstrated that with fewer than 40 annotated Mass2Motifs a partial annotation of >70% of the metabolite features in beer extracts could be made^16^ – showing the promise of a substructure-based metabolomics approach. Another use case is to start from the discovery of yet unknown or unexpected substructures through their fragmentation patterns that could lead to the characterization of novel metabolites: for example, guided by MS2LDA analyses, the first examples of hybrid alkylated phenylpropane monoterpene indole alkaloids were isolated from *Callichilia inaequalis* through the annotation of a phenylpropane-related Mass2Motif.^19^ Furthermore, in Fig. 2, it can be seen how MS2LDA could identify three main features from the MS/MS spectra of these polyphenol metabolites. The main Mass2Motif isoscutellarein is indeed present in all the metabolites depicted on the figure, as well as the glucose feature. The acetate is clearly identified on the acetylated compounds resulting from the transformation of the compounds on the bottom left corner, illustrating how MS2LDA supports the metabolite annotation process and how MS2LDA can discover Mass2Motifs that are directly related to (plant) biosynthesis.

In the original MS2LDA pipeline, all Mass2Motifs need to be annotated by researchers using expert knowledge or through fragment-based searches in spectral libraries. Furthermore, each analysis would learn a complete set of new Mass2Motifs. However, the same Mass2Motifs will be rediscovered when similar sample types are analysed, motivating the development of an open database of structurally characterized Mass2Motifs (MotifDB^20^) to which any user can add newly annotated Mass2Motifs – known as MotifSets. Where a MotifSet was measured from similar samples in the same ionization mode, it can be included in a new MS2LDA analysis. MS2LDA will extract these Mass2Motifs simultaneously with learning new Mass2Motifs, accelerating the annotation process by simultaneously acknowledging known substructures and considering novel chemistry. MS2LDA is available in the GNPS^21^ molecular networking pipeline as well as through a dedicated web application,^22^ at which MotifDB is currently also hosted.

### 2.3 Substructure recommendation by MESSAR

A complementary approach to MS2LDA substructure discovery is the MEtabolite SubStructure Auto-Recommender (MESSAR). MESSAR was developed to recommend substructures that are likely to be present in unlabeled (unannotated) MS/MS spectra.^23^ This is inspired by recommendation services that suggest purchases or services based upon individuals' previous behaviour and choices. To train MESSAR in recognizing the potential relationships between spectral features and substructures, GNPS public spectral libraries^21^ were used. These annotated spectra (with known molecular structure) were used as reference spectra for establishing links between mass spectral features (mass fragments, neutral losses, and mass differences) and substructures present in the molecules. The latter are defined by determining the molecular fingerprints present from the SMILES representations of the molecules, resulting in vectors where the value in a particular position indicates whether or not a particular substructure is present (*i.e.*, aromatic ring or nitrogen-containing 5-membered ring). Then, rules are established to connect these substructures to the mass spectral features derived from the MS/MS spectra, thereby also using the hypothesis that the same substructure produces the same or similar mass spectral features independent of the other parts of the molecule that are connected to the substructure.

The approach is inspired by the concept of association rule mining (ARM) that discovers interesting relations based on frequently co-occurring items. In the training process, a database of 8378 mass spectral features to substructure rules was established. To benchmark and validate their approach, a comparison with MS2LDA was performed on the same set of GNPS library spectra in which a number were previously validated using expert knowledge.^16^ A reliable overlap between the 8378 MESSSAR rules and 77 M2Ms (out of 500) was found, with the annotations being identical or very similar to MESSAR substructure recommendations for 26 out of 28 previously validated Mass2Motifs. MESSAR is available as a web-based tool. Overall, MESSAR and MS2LDA are complementary approaches, since the rule-based approach and topic modelling substructure discovery work differently: the learnt MESSAR rules connect mass spectral features with specific substructures, while Mass2Motifs are spectral substructure patterns derived from raw experimental spectra and can be learnt for completely unknown chemistry as well, whereas MESSAR rules need to be established on library spectra with known structures and mass spectra. The authors also show strong orthogonality between MESSAR, MS2LDA, and CSI:FingerID (discussed in the next section) in terms of the substructure types these tools accurately annotate: MESSAR performed best for polycyclic aromatics, indoles, and chlorobenzenes, MS2LDA for sterones and sugar conjugates, and CSI:FingerID for amino acids and benzenesulfonyl amides. Given the complementary nature of these approaches, we imagine that a meta-approach that integrates their outputs could further increase substructure annotation accuracy. The annotated MotifSets from MotifDB can also be obtained through an Application Programming Interface (API) and having a similar service available in the future for MESSAR would further increase its options to integrate it into existing and future pipelines. For example, this would allow incorporating the most confident recommended substructures into an annotation pipeline for automated Mass2Motif substructure pattern annotations.

### 2.4 Molecular fingerprint-based metabolite annotation by CSI:FingerID

*In silico* annotation of MS/MS spectra with molecular structures in the absence of reference spectra typically adopts one of two core strategies. In the first, spectra predicted from candidate structures are compared with observed spectra.^24,25^ In the second, structural properties (normally molecular fingerprint vectors) are predicted from the observed spectra and compared with the same properties derived from candidate structures. Of these, the latter approach has historically outperformed the former.^26^ Of the fingerprint-based approaches, CSI:FingerID, part of the SIRIUS software package^27^ is the state-of-the-art.

CSI:FingerID starts with the computation of fragmentation trees from the MS/MS spectra in which each mass fragment becomes a tree node with the precursor ion as the tree origin and the connections in the fragmentation tree representing small chemical modifications that are assumed to be part of fragmentation pathways occurring in the collision cell of the mass spectrometer. A number of connected and thus related tree nodes typically represent a substructure of the fragmented molecule. This approach has shown enormous gains in accurate elemental formula assignments,^27^ and was recently complemented with a network strategy to also gain improvements for large molecules (>500 Da).^28^ The fragmentation tree forms the input to a machine learning method for fingerprint prediction. Here, CSI:FingerID makes use of a kernel method: a class of machine learning methods that have been shown to perform well across many domains. The Support Vector Machine (SVM; see *e.g.*^29^) is the most popular kernel-based classifier, and is at the heart of CSI:FingerID. CSI:FingerID uses multiple SVM classifiers to predict, from the fragmentation tree, the presence or absence of each of a set of several thousand fingerprint elements resulting in a vector of the probabilities of presence of each fingerprint element, *i.e.*, the presence/absence of an aromatic ring, presence/absence of a nitrogen atom, *etc.* From compound databases, having their structures in hand, the same fingerprint properties can be derived for the candidate structures obtained; and therefore, these candidates can be ranked according to the similarity of their fingerprint vectors with that predicted from the query spectrum. A key feature of SVMs in this context is that they do not explicitly use the fragmentation tree to make predictions, but rather the similarity between pairs of trees. That enables the combination of multiple similarity measures (*via* kernel functions) to allow for the leverage of different representations of the input data. Indeed, CSI:FingerID uses different types of structural information derived from the computed fragmentation trees for its predictions.

## 3 Chemical class-based MS-based metabolomics tools

### 3.1 Chemical classification of metabolomics features

The annotation of entire metabolite structures is very challenging and substructure-based strategies as described above are thus an attractive route to decipher complex metabolite mixtures. An alternative is to annotate metabolite features at the chemical compound class level. Chemical compound classes such as flavonoids, polyketides, and peptides comprise many different structures that do have some structural elements in common. Many of these classes have been historically defined, often based on a combination of structural and functional properties. In that sense, chemical compound classes could be regarded as a special variant of substructures where sometimes multiple substructural features together with a specific biological activity define a compound class. For example, pharmaceutical activities (antivirals, antihypertensives) and the biosynthetic origin (nucleic acids, terpenoids) have been used. Over time, many classes have been split into subclasses as well; for example, flavonoid-3-*O*-glycosides is considered a subclass of the flavonoids. If all metabolite features measured in complex metabolite mixtures could be annotated at such chemical compound class or subclass levels, researchers would be able to focus on a subset of metabolite features that belong to the chemical class that they study, for example, or investigate those that are likely to be completely novel. Indeed, for a number of compound classes targeted or semi-targeted approaches have been proposed, mostly based on the presence of specific mass spectral features in the mass fragmentation spectra; however, only since the last couple of years, various approaches have been introduced that are able to link chemical class annotation to large-scale metabolomics analyses. One key element that enabled these developments was the introduction of chemical ontologies, in particular those that can be directly linked to and determined from textual representations of metabolite structures. The latter allows computational workflows to classify candidate structures for mass spectral features or to train machine learning models to recognize the links between MS/MS spectra and chemical class terms, instead of the manual inspection of the metabolite structures or usually semi-automated extraction of key characteristic mass spectral features from MS/MS spectra otherwise needed. Furthermore, such a strategy can target the large variety of chemical structures that complex metabolite mixtures typically contain. In this section, we will highlight important chemical ontologies and currently available methods to perform large-scale chemical compound class annotations as part of untargeted metabolomics workflows.

### 3.2 Chemical ontologies and taxonomies

#### 3.2.1 ChEBI

Ontologies and taxonomies are of great value across many scientific disciplines as they help scientists to organize complex knowledge about concepts and to define their relationships. Taxonomies are schemes that establish hierarchical classification of concepts or objects. Ontologies share the hierarchical structure of taxonomies, but often allow for multiple relationship types and they introduce a formal naming of the types, properties and interrelationships of entities or concepts. One of the first extensive structural ontologies that include chemical compound class annotations is part of the Chemical Entities of Biological Interest (ChEBI) database.^30^ In their Chemical Ontology, the molecular structure sub-ontology classifies molecular entities or parts thereof according to elemental composition and structure, *e.g.*, hydrocarbons, carboxylic acids, or tertiary amines. This manually curated ontology has the form of a directed acyclic graph with also some cyclic relationships, thus meaning that a child term can have many parents. For example, quercetin 3,4′-di-*O*-β-d-glucoside has many direct parent terms including monosaccharide derivative, β-d-glucoside, polyphenol, and trihydroxyflavone, each describing a different aspect of the natural product in a more generic or specific manner. One can follow these terms all the way up toward “chemical entity”, for example, polyphenol – phenols – organic aromatic compound – aromatic compound – *etc.* The LIPID MAPS comprehensive classification system for lipids is worth mentioning here as well as an example of such an ontology system focusing on lipid molecules.^31^ Whilst these ontologies have been applied successfully over the years, the manual curation does make the classification and annotation process quite tedious and sometimes inconsistent whilst also requiring a lot of human expert knowledge.

#### 3.2.2 ChemOnt & ClassyFire

To overcome the limitations of the ChEBI molecular structure ontology and enable large-scale automated chemical class annotations of molecular structures, the ChemOnt ontology was introduced.^32^ ChemOnt is ClassyFire's comprehensive chemical taxonomy currently covering 4825 chemical classes of organic and inorganic compounds to robustly characterize, classify and annotate chemical structures. ClassyFire was the first automated tool to add chemical class annotations to candidate structures using as input their textual representation (using SMILES^33,34^ or InchiKeys^33^). Upon submission of a structure, ClassyFire returns a hierarchical compound class annotation based on the presence and absence of particular substructures using the SMiles ARbitrary Target Specification (SMARTS) format to detect them. Assigned chemical taxa terms include many terms relevant for natural product chemistry including triterpenoid, flavonoid, or hydrolysable tannin but also cover primary metabolites such as those found in urine since the ChemOnt ontology primarily serves generic metabolomics research. For example, ClassyFire returns for quercetin 3,4′-di-*O*-β-d-glucoside the following terms: kingdom: organic compounds – superclass: phenylpropanoids and polyketides – class: flavonoids – subclass: flavonoid glycosides – intermediate tree nodes: flavonoid *O*-glycosides – direct parent: flavonoid-3-*O*-glycosides. In the ChEBI ontology, comparable terms are part of the ontology tree for this compound, whilst ClassyFire also contains terms like phenolic glycosides as alternative direct parent; however, the flavonoid related path was deemed more relevant by ClassyFire. With the continuous discovery of novel chemical structures, we hope that the ChemOnt ontology and the associated classification rules will be extended and adapted over time to reflect new knowledge.

#### 3.2.3 NPClassifier

Although ClassyFire and CheBi paved the way to automated class annotations of chemical structures, not all natural products seemed to fit in well in their classification systems. The most important reason is that natural products typically have a slightly different criterion of classification than other chemicals. General chemical classification systems classify each compound based on structural properties (*e.g.*, functional groups) of the compound itself; however, the historical natural product classification system has mostly been generated based on how the compound was generated in the organismic system: the biosynthetic pathway. Thus, NPClassifier^35^ was proposed to link historical natural product classifications to structures using deep learning. NPClassifier converts the structure provided as a SMILES to a chemical fingerprint, then classifies it with a deep neural network model built with 73 607 structures collected from public databases and the three-level ontology system organized as 7 pathways, 70 superclasses, and 653 classes defined based on literature search. Although this tool has been just introduced to the community, it is expected to enhance a number of computational biology pipelines for linking genome and metabolome datasets. Finally, it is presented as an open access tool to the community and novel natural product compound classes can easily be trained with sufficient available examples and added to the classification scheme.

#### 3.2.4 CANOPUS

The first tools that build upon these chemical ontologies have emerged. MolNetEnhancer^36^ uses the ChemOnt ontology to provide a higher-level chemical overview and is covered in Section 4.2.4. More recently, CANOPUS^37^ was introduced which uses a deep neural network to predict 2497 ClassyFire compound classes from fragmentation spectra. This workflow does not depend on annotated candidate structures but can assign compound classes directly to MS/MS spectra as long as a fragmentation tree can be computed (see also Section 2.4 on CSI:FingerID). It is expected that NPClassifier terms will be added to CANOPUS in the near future.

## 4 Network-based MS-based metabolomics tools

### 4.1 Grouping metabolite features based on mass spectral similarity

This section describes recent key tools that group mass fragmentation spectra based on their mass spectral similarity to form networks of the fragmented metabolite features. Such molecular networks or mass spectral networks help to logically organize the large number of mass fragmentation spectra now typically obtained within metabolomics experiments thus supporting biochemical interpretations. Different approaches are currently available to the metabolomics researcher and are widely used by natural products researchers. Here, we highlight and explain some of the currently most widely used ones as well as some emerging tools that rely on input from molecular networking. We use a case study of Monoterpene Indole Alkaloids to show how mass spectral networking can aid in biochemical interpretations but is also dependent on the mass spectral similarity metrics and thresholds used to form the network. The section ends with a brief overview of current tools that allow networking analysis on gas chromatography (GC-)MS datasets using electron impact ionization. This extends networking-based analyses to volatile measurements, another important group of molecules not covered by LC-MS metabolomics measurements.

#### 4.1.1 Molecular networking

First introduced in 2012,^38^ molecular networking (or more generically called mass spectral networking) has since become a popular tool in the analysis of MS/MS-based metabolomics data.^21,39^ The theoretical rationale of the method was quite straightforward. As fragmentation spectra acquired in MS/MS analyses are hypothesized to be related to their original chemical structures, molecules with similar structures will exhibit similar MS/MS spectra. Thus, if we can calculate spectral similarities between all spectra within a complex mixture, the spectral similarities can be extrapolated to the structural similarities between molecules in the mixture. The matrix of spectral similarity can be further visualized as a graph called a spectral network or a molecular network, where each node is a MS/MS spectrum, and edges between nodes indicate spectral similarity above the similarity score threshold defined by the user. The mass spectral similarity is calculated with a modified cosine score. Each MS/MS spectrum is simplified as a vector in a multidimensional space where each dimension corresponds to an *m*/*z* value of fragment ion and its ion intensity. Then the angle between two spectral vectors in the space is calculated to express the similarity between two spectra. This is the cosine score, but Global Natural Products Social (GNPS) molecular networking put a subtle modification to the algorithm. Peaks from one spectrum are aligned with peaks from the other either in their original *m*/*z* position or with their *m*/*z* shifted according to the difference in the precursor *m*/*z* of the two molecules. The rationale behind this is that a single modification to a structure will often lead to a spectrum in which a subset of the fragment peaks has shifted by the *m*/*z* shift of the modification (*e.g.*, −18 Da for a water loss). The theoretical background of the method may sound simple, but its introduction caused a paradigm shift in the analysis of MS/MS spectra. The beginning of a network analysis approach means relationships between the spectra started to be considered, while each spectrum was analyzed independently in conventional pipelines. By grouping spectra based upon their similarity, molecular networking allows identifying so-called molecular families corresponding to communities (or clusters) in network theory.^40^ As molecular families are clusters of molecules whose structures are expected to be similar to each other, it gives multiple advantages to further metabolomics data analysis. At first, structural information on any molecular family member could be propagated into other family members. Network Annotation Propagation (NAP)^41^ automates this process by exploiting this grouping to re-rank candidate structure annotations from compound databases considering the consistency of the structures within a molecular family: *i.e.*, if 8 out of 10 nodes have a flavonoid glycoside as top ranked candidate, then the slightly lower ranked flavonoid glycoside structures for the two remaining nodes are more likely to be the correct annotation than the top-1 ranked candidates that do not have structural resemblance to flavonoids (see also Section 4.2.2). For an extensive review of the various tools that have been integrated within the GNPS platform in the context of natural products research, we refer to Fox Ramos *et al.*^39^ Another important advantage molecular networking provided to the community is that it allowed chemically-informed comparative analysis between samples. Multivariate methods conventionally used in metabolomics treated every spectral feature as being orthogonal to each other. However, as metabolites are products of biological reactions, chemical relationships between each molecule should be in consideration. Molecular networking allows such chemically informed sample-to-sample comparison, even in cases where any spectral member of the molecular family is not identified, as shown in development of the chemical structural compositional similarity^42^ and Qemistree which is further covered in Section 8.4.^43^

#### 4.1.2 Alternative similarity scores and visualization for mass spectral networks

As highlighted above, mass spectral similarity is used as a proxy for structural similarity in MS/MS-based network analysis. Although mismatches between spectral similarity scores and the true structural similarities are frequently observed, little development of alternative scores has been undertaken. It is therefore very promising to see the recent development of a novel mass spectral similarity score based on an unsupervised machine learning approach inspired by the natural language processing algorithm Word2Vec,^44^ called Spec2Vec.^45^ Spec2Vec learns fragmental relationships within a large set of spectral data to derive abstract spectral embeddings that can be used to assess spectral similarities. Using data derived from GNPS MS/MS libraries including spectra for nearly 13 000 unique molecules, it was demonstrated how Spec2Vec scores correlate better with structural similarity than cosine-based scores especially for structures that are not fully identical but share most of their structural features. Consequently, a higher accuracy was reported for library matching experiments. Furthermore, the Spec2Vec score was used for mass spectral network analysis as well as large-scale analogue search where a large database is searched for structurally similar molecules based on their MS/MS spectra without using any parent mass filtering. To calculate the Spec2Vec scores, a mass spectral embedding needs to be learnt once. Subsequent mapping of new experimental MS/MS spectra on this embedding is then very fast. As a consequence, Spec2Vec returns structural analogues found in large databases within seconds, with particularly good results for molecules in the higher mass range (400–2000 Da) as shown for a GNPS library of >75 000 spectra with a cyclopeptide and lipid example.^45^ We expect that Spec2Vec will trigger the emergence of more novel machine learning-based mass spectral similarity scores, both unsupervised and supervised, to further improve its performance for a range of diverse tasks such as the described library matching and analogue search, but for example also adduct annotation and mass spectral network creation tailored toward improved resolution for specific compound classes. In fact, the first example of a supervised machine learning-based approach was just proposed.^46^ These developments may further assist in the biochemical interpretation of such mass spectral networks thereby facilitating the process of turning large-scale untargeted mass spectral analyses into biochemical knowledge.

Olivon *et al.* developed MetGem,^47^ a software by which molecular networks can be generated based on two different algorithms: cosine similarity between aligned spectra (the one used in the GNPS molecular networking) and t-SNE (stochastic neighbour embedding) algorithm, a well-known technique used for high-dimensional data visualization.^48^ The t-SNE based graph does position spectra due to local details within the entire data space, which makes it possible to also draw conclusions about inter-cluster relations (*e.g.*, based on their distance) for closely related clusters. It further allows clustering spectra without relying on individual links between spectra and can thereby avoid the tradeoff between having too many non-connected nodes or fusing clusters which occurs when setting a similarity cutoff in molecular networking. However, t-SNE does not provide information on the relationships between individual spectra, which could then be obtained by applying additional workflows such as NAP annotation and meta-mass shift analysis.^49^ Thus, the t-SNE-based visualization and clustering is a method complementary to the cosine similarity-based clustering.

#### 4.1.3 Case study: molecular networking with monoterpene indole alkaloids

**Fig. 3:**
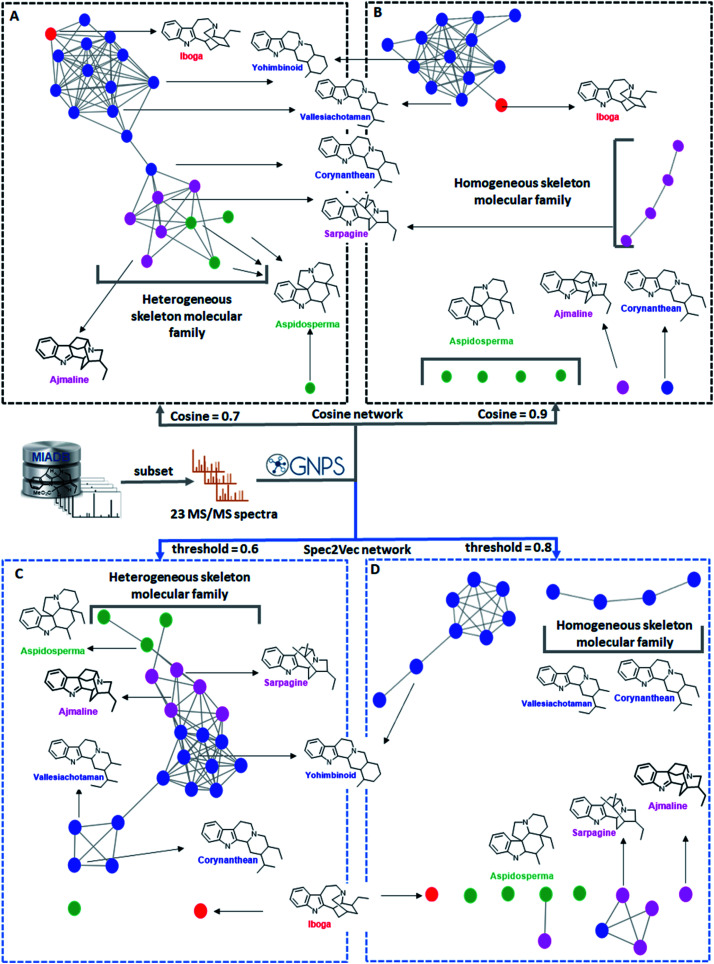
Molecular networks of 23 monoterpene indole alkaloids generated using two different cosine score values (panels A and B) and two different Spec2Vec threshold scores (panels C and D) and color-tagged according to different categories based on their manually curated biosynthetic scaffolds with related scaffolds tagged in the same color. The seven representative skeletons are displayed with their names colored according to these categories. (A) Molecular network created with a modified cosine score threshold of 0.7, displaying one molecular family constituted by various skeletons and one singleton. (B) Molecular network of the same 23 nodes but at a modified cosine score threshold of 0.9, featuring two homogeneous molecular families and six singletons. (C) Spec2Vec-based molecular network using a Spec2Vec score threshold of 0.6, displaying one molecular family constituted by various skeletons and two singletons. (D) Spec2Vec-based molecular network using a Spec2Vec score threshold of 0.8, displaying three molecular families constituted by homogeneous skeletons and five singletons. We demonstrate how the use of different mass spectral similarity metrics and score thresholds results in different network topologies and thus influence the biochemical interpretability.

Monoterpene Indole Alkaloids (MIAs) represent a complex structural family of more than 3000 (ref. 50) compounds featuring an impressive array of structural variants which are divided into 42 representative skeletons. In addition, MIAs are also known for their various isomeric possibilities leading to analytical intricacies with respect to their annotation. To gain insight in how well network analyses reflect the – sometimes subtle–structural relationships of the MIAs, we created modified cosine (as used in GNPS) and Spec2Vec-based mass spectral networks. To illustrate the successes and the limitations of network analysis, 23 selected MS/MS spectra derived from the monoterpene indole alkaloid database (MIADB)^51^ and belonging to seven representative skeletons (Fig. 3), were subjected to the classical molecular networking workflow using two different modified cosine score thresholds (Fig. 3A and B) and two different Spec2Vec thresholds (Fig. 3C and D). For this case study, compounds belonging to the yohimbinoid, corynanthean, and vallesiachotaman skeletons are considered as structurally and biosynthetically related scaffolds and were all color-tagged in blue, since they share the same indole-quinolizidine sub-structure. The same reasoning applies to the compounds pertaining to the ajmaline and sarpagine skeletons, as they share the bridgeheaded-indole–quinolizidine motif. These two skeletons were coloured in purple. As depicted in Fig. 3A, a modified cosine score threshold of 0.7 resulted in one selfloop node in addition to one molecular family that brought together six various MIA skeletons (*i.e.*, iboga, yohimbinoid, corynanthean, vallesiachotaman, sarpagine, aspidosperma, and ajmaline). With these parameter settings and threshold, the singleton metabolite (19-acetyltabersonine), despite being related to the aspidosperma skeleton (tagged in green), could not be linked to its three analogues, namely, tabersonine, 16-hydroxytabersonine, and 19-hydroxytabersonine. It is interesting to note that in the large molecular family, the three distinct skeleton families are grouped together, indicating that the network topology does reflect to some extent that these structures are more related to each other than to other members of the molecular family. In Fig. 3B, we observe how a higher threshold value led to the generation of two molecular families of structurally similar compounds along with six selfloops. Satisfyingly, the four compounds related to the sarpagine skeleton were linked together in this stricter clustering. However, the four above mentioned aspidosperma-related compounds were scattered as singletons. In both cases, the iboga skeleton catharanthine (coloured in red), which is present here as a sole representative, failed to be organized as a singleton and, systematically, ends-up connected to yohimbinoid, corynanthean and vallesiachotaman skeletons, despite its apparent structural dissimilarity. In contrast, catharanthine has been successfully depicted as a singleton in both Spec2Vec networks (Fig. 3C and D). In addition, it is worth noting that the yohimbinoid skeleton was nicely distinguished from the other indole–quinolizidine-containing skeletons (*i.e.*, corynanthean and vallesiachotaman) (Fig. 3D). Altogether, this case-study highlights the successes and the limitations of both spectral similarity algorithms in linking apparently structurally similar yet subtly different molecules from various biosynthetic origins. This example also illustrates the difficulties in inferring structural similarity from mass spectral similarity. The observed discrepancies could well stem from fragmentation rules occurring in the gas phase that do not always allow us to discriminate well based on the biosynthetic skeleton classification. A possible explanation is that in some cases minor differences in the structure, *i.e.*, the addition of a methyl or hydroxyl group, can substantially change the preferred fragmentation paths leading to different diagnostic fragments of the same skeleton. As such, it is promising to see the emergence of Spec2Vec^45^ mass spectral similarity scores performing complementary to the existing widely used cosine-based scores. In the future, this may help us to find alternative ways to link the spectra of closely related terpene and alkaloid analogues such as the MIAs studied here.

#### 4.1.4 Molecular networking with electron impact (EI) ionization MS data

Early efforts on networking analysis with MS data were focused on electrospray ionization (ESI), which is a common choice for hyphenation with HPLC. Electron Impact (EI) MS, which is generally coupled to GC, is another important domain of MS-based metabolomics that measures volatiles or derivatized molecules that can also have important functions, so efforts to establish molecular networking workflows for EI MS have started. The first EI MS-based molecular network was established with MetGem,^52^ together with MZmine2.^53^ In that study, spectral deconvolution was performed using hierarchical clustering in MZmine, then parent mass information was removed from all the MS features; this processing allowed the software to build molecular networks based on only fragment ions, not neutral losses. Approximately one year later, the GC-MS-based molecular networking workflow was introduced in GNPS.^54^ The workflow performs auto-deconvolution of compound fragmentation patterns *via* unsupervised non-negative matrix factorization, using a fast Fourier transform-based strategy to overcome scalability limitations. A “balance score” has been introduced in order to quantify the reproducibility of fragmentation patterns across all samples.

### 4.2 Annotating the metabolite features in the network

#### 4.2.1 Spectral library matching

The most common and reliable method for metabolite annotation (where an MS2 spectrum is available) is through spectral library matching. Here, observed spectra are compared against spectra stored in reference libraries (*e.g.*, MassBank, METLIN, GNPS, *etc.*). For a particular observed query spectrum A, reference spectra are typically first filtered to only include those with a precursor *m*/*z* sufficiently similar to that for A. The remaining spectra are then scored against the query, most often based upon some variant of the cosine score (the inner (dot) product between two vectors, normalized to lie between −1 and 1). As spectra don't naturally lend themselves to points in a vector space, either the *m*/*z* values are binned and discretized into a fixed length vector or an alignment step is performed in which *m*/*z* peaks are matched between the spectra. Because the intensity values in a spectrum have to be positive, cosine scores for pairs of spectra should always lie between 0 and 1.

Various variants on the cosine score are used. These differ by (a) the ways in which the input spectral intensities are normalised, (b) whether or not the *m*/*z* as well as intensity is explicitly used in the calculation (*e.g.*, to upweight contributions for heavier (and therefore rarer) peaks). Forward and reverse cosine scores can also be used which can be interpreted as measuring how much of one spectrum exists in the other (*i.e.*, how much query can be found in reference, or how much reference can be found in query). Other variations include the modified cosine (see Molecular networking section above) in which fragment peaks can be shifted by the *m*/*z* difference of precursors, to account for simple chemical modifications.

Although spectral search is the most popular and reliable method for annotating metabolite features, challenges still exist. The most pressing is the relatively small size of the reference databases: only a very small subset of known chemicals are covered. In addition, although work has been done in this area,^55–58^ there is still no widely used method for computing false discovery rates for these data, which can make interpretation of cosine scores highly subjective without extensive manual inspection of spectral matches in terms of overlapping peaks and their intensity patterns.

#### 4.2.2 Structure libraries for structural annotation

Although the number of mass spectra deposited in spectral libraries are growing fast, it is still much less than the total number of known chemicals. *In silico* fragmentation tools are one of the solutions to overcome this limitation of spectral library matching. By taking advantage of structural libraries, computational fragmentation tools increase the annotation rate in MS/MS-based metabolomics studies.^2^ Since metabolite identification has been a bottleneck in untargeted metabolomics pipelines, various *in silico* tools have been developed: CSI:FingerID^59^ and DEREPLICATOR+ (ref. 60) are recent examples.

As molecular networking has risen as one of the major methodologies in metabolomics, integration of *in silico* methods with molecular networking has been explored. ISDB-DNP is a hypothetical spectral database which was generated by CFM-ID^24^ with structures from *Dictionary of Natural Products*;^61^ and it was successfully integrated with molecular networking to provide metabolite annotations.^62^ More recently, taxonomically informed scoring was suggested as a method for enhancing the confidence of annotation using ISDB-DNP for natural product datasets.^63^ Network Annotation Propagation (NAP) is the first *in silico* annotation method which directly exploits the network topology provided by molecular networking.^41,64^ It re-ranks the candidate structures found by MetFrag^41,64^ based on the expected consistency of annotations within molecular families of connected components (see Section 3.1).

#### 4.2.3 MetWork

MetWork^65^ was designed as a tool for specialized metabolite “anticipation”. The main idea of anticipation is that all the molecules of a metabolome are connected by at least one chemical or biochemical transformation.^66,67^ It represents an implementation of the virtuous circle of metabolite identification^68^ and is composed of three modules: a participatory database containing the available chemical transformations, a spectra prediction module based on CFM-ID^24^ and a module for comparing predicted and experimental spectra allowing the annotation of molecules. This web service has been used in several studies leading to the identification of new compounds using the logical link which are biosynthetic transformations to locate and name interesting ions in the extracts. The whole process of natural products anticipation has been further formalized into the computer assisted natural products anticipation (CANPA) approach,^69^ which is giving more weight to the links between compounds that were previously reported. Following the CANPA approach, five new sarpagine *N*-oxide alkaloids were discovered starting from known sarpagine alkaloid structures that were *in silico* biotransformed using reactions including, *N*-, and *O*-methylation, *N*-oxidation, and *para*-indole hydroxylation known to occur for these alkaloids. The new structures were isolated, and their postulated structures could be verified with NMR. It is clear that this approach relies on known structures to serve as known anchors in the network, and a good dereplication pipeline is thus mandatory for the proper functioning of the MetWork tool. Altogether and following the computer assisted natural products anticipation (CANPA) approach, MetWork helps to move from the collection of data and information toward the creation of knowledge in the data, information, knowledge, and wisdom (DIKW) pyramid.^69^

**Case study: identification of flavone glycosides from *Sideritis hyssopifolia***. The combination of both MetWork and MS2LDA for the annotation of several flavone glycoside analogues from the plant *Sideritis hyssopifolia* is shown in Fig. 2. Using the compounds from the literature (green nodes) as an input for the *in silico* metabolism gives rise to multiple putative compounds using simple transformations such as ester hydrolysis or methylation (blue rectangles). The prediction of MS/MS spectra corresponding to the putative compounds is used as a threshold to allow an annotation of the proposed structures. For this specific class of compound, the comparison between theoretical and experimental MS/MS spectra using the dot product metrics provides good agreement (values > 0.58). Further confirmation of the compound identification is given by MS2LDA. The modifications made through *in silico* metabolism are well identified by the specific neutral losses and fragments on the MS/MS spectra (bottom right corner in Fig. 2).

#### 4.2.4 MolNetEnhancer

The multitude of mass spectral metabolome mining and annotation tools and manifoldness of different output formats and analysis platforms hamper the easy visualization of output from complementary tools within one data file or platform. This realisation has driven the development of MolNetEnhancer.^36^ MolNetEnhancer is a workflow that combines the output from spectral library matching and *in silico* structural annotation onto a GNPS mass spectral molecular network. In this way it not only facilitates analysis, but also allows for powerful visualizations of the chemical diversity in the dataset. Chemical class annotation takes place by calculating the most predominant chemical classes retrieved for all top candidate matches per molecular family at each hierarchical level of the ClassyFire chemical ontology onto the network. Finally, substructure motifs (Mass2Motifs) learnt by MS2LDA that are shared between mass features of the same molecular family are visualized through additional edges connecting the nodes. Thus, instead of comparing tables and output from multiple platforms, information from mass spectral molecular networking, *in silico* structure annotation and substructure discovery can easily be visualized within one datafile in Cytoscape.^70^ This allows researchers to investigate and strengthen structural hypotheses by collecting information from several complementary metabolome mining tools. Recently, MolNetEnhancer was used to demonstrate that sesquiterpene lactones, flavonoids, fatty acids, and fatty acid amides of various chicory (*Cichorium intybus* L.) cultivars displayed bioactivity against the parasitic helminth *Ascaris suum*.^71^ Furthermore, the workflow was used to assess the wide range of natural products of the filamentous fungus *Paecilomyces* sp. CMAA1686 isolated from a cemetery in Brazil, which includes pharmacologically active scorpionicidal (against *Tityus serrulatus*) terpene lactones, phenylpropanoids, and alkaloids.^72^ MolNetEnhancer can conveniently be run through the GNPS platform,^21^ currently supporting *in silico* structural input from DEREPLICATOR,^73^ a tool to annotate peptidic natural products, and Network Annotation Propagation.^41^ In the future, it is anticipated that the output of other metabolite annotation tools such as DEREPLICATOR+,^60^ which annotates besides peptides also other compound classes such as polyketides and flavonoids, and CANOPUS^37^ will also be compatible with the MolNetEnhancer workflow.

## 5 Substructure discovery by NMR

**Fig. 4:**
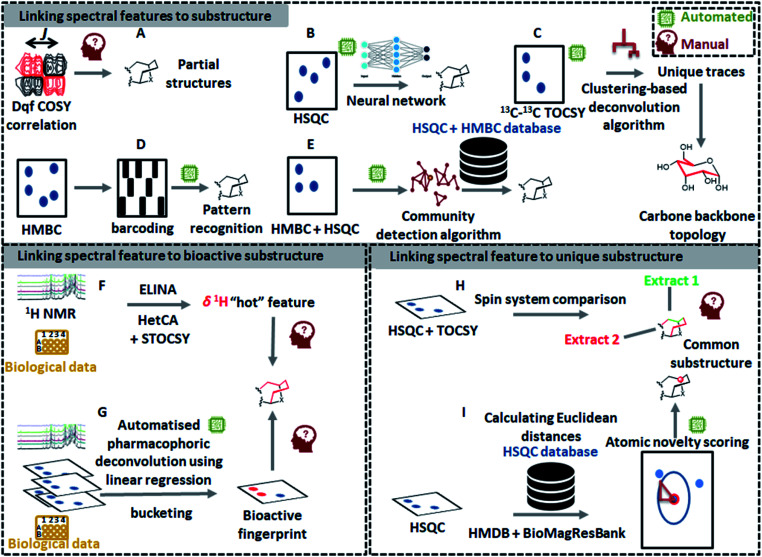
An overview of existing manual and automated 1D-NMR and 2D-NMR concepts for chemical classification and/or substructure finding. The top panel shows how pattern recognition ((A) see 5.1.1, (C) see 5.1.4, (D) see 5.1.2, (E) see 5.1.3) and machine learning approaches ((B) see 5.1.5) allow to extract NMR spectral features that are linked to substructures. Automated workflows start to emerge for the various types of 2D-NMR experiments; thus, manual input and validation remains important as 2D-NMR peak picking can still be tricky and challenging and is thus often a manual task. To prioritize NMR spectral features in complex datasets for further analyses and elucidation, methods to link them to bioactivity (panel at lower left: (F) see 5.2.1, and (G) see 5.2.2) and to assign them a novelty score (panel at lower right: (H) see 5.3.1 and (I) see 5.3.2) are also emerging. We expect that with the increase in available NMR data and computational metabolomics approaches, the above-shown concepts will further mature to come to (nearly) fully automated pipelines for NMR-based substructure and chemical class assignments.

The latest developments in nuclear magnetic resonance (NMR) spectroscopy experimentation and instrumentation have led to a significant increase in the use of NMR-based metabolomics for the dereplication of natural products.^14^ Recently, several innovative 1D NMR-based tools have emerged such as MixONat^74^ and CARAMEL,^75^ just to name a few; however, the identification of compounds within complex mixtures using NMR spectroscopy is still challenging mainly due to NMR signal overlap that masks lower abundance signals and distorts signal patterns and their quantitative areas.^74–76^ As a way to overcome these resolution issues, deconvolution of signals has been considered to be an essential step for NMR-based identification of metabolites in complex mixtures. As such, Diffusion Ordered SpectroscopY (DOSY)^77^ and Statistical TOtal Correlation SpectroscopY (STOCSY)^78^ are key methods for spectral deconvolution in NMR-based metabolomics. DOSY utilizes diffusion coefficients of molecules, while STOCSY calculates correlation coefficients between all the resonances across the entire mixture data. Since the first introduction of STOCSY, several adaptations have been proposed to improve its performance: such as Peak Overlap Detection by Clustering Analysis and Sorting of Traces (POD-CAST)^79^ and COrrelation COmparison Analysis for Peak Overlap Detection (COCOA-POD).^80^ Meanwhile, another approach for NMR spectral deconvolution using ^13^C–^13^C NMR correlation spectrum and indirect covariance eigendecomposition was introduced recently, which successfully deconvoluted ^13^C spectra of rotenone and brucine from the spectrum of their mixture as a benchmark.^81^ However, all of these methods only deconvolute the complex spectra; manual interpretation of NMR spectra is still required to identify the chemical structures. Compared to MS, only a few automated NMR-based tools have been developed for substructure discovery from complex mixtures. Yet, some recently developed technologies, dedicated to the structural recognition of molecules, can be exploited to recognize partial spectral features that correspond to substructures in the measured molecules. In the following sections, different ways of detecting substructures by NMR will be outlined (Fig. 4). The first one will describe the approaches that enable substructure assignment starting from spectral features, the second will describe the tools that link substructure fingerprints to their function (*i.e.*, biological data), whereas the last one will present the tools that allow to detect spectral features that can be linked to unusual (and often previously unseen) substructures.

### 5.1 Linking spectral features to substructures

#### 5.1.1 dqfCOSY: generation of partial-structures from crosspeaks and pattern recognition

Double-quantum-filtered correlation spectroscopy is particularly suitable for the analysis of complex small-molecule mixtures because it displays detailed coupling constant information, and often permits clear recognition of long-range ^1^H–^1^H-couplings, and displays easily modeled cross-peak patterns that frequently enable interpretation of overlapping signals. Schroeder *et al.* used high-resolution dqfCOSY to screen a library of bio-rationally selected insect extracts to manually generate libraries of structural fragments (“partial structures”)^82^ based on spin system connectivity. Further analysis of the data obtained of secretions from pupal *Delphastus catalinae* data revealed a large group of polyketide-like fragments. Satisfyingly, the subsequent database searches for these motifs and associated NMR-spectroscopic data indicated that these compounds were unprecedented in nature. Based on this observation, this insect was selected for further study and this led to the discovery of catalipyrones, ten effective insect-repellent polyketides featuring an unusual 23-carbon skeleton.

#### 5.1.2 HMBC barcoding

Pauli *et al.* developed an innovative concept of 2D-NMR barcoding that uses clusters of fingerprint signals and their spatial relationships in the *δ*_H_–*δ*_C_ coordinate space to facilitate the chemical identification of complex mixtures.^83^ The structural information of individual compounds is encoded as a specific pattern of their carbon–proton correlation signals. Software-based recognition of these patterns enables the structural identification of the compounds and their discrimination in mixtures. This approach was applied to explore the triterpenes from various *Actaea* (syn. *Cimicifuga*) species as a test case. Heteronuclear multiple-bond correlation (HMBC) barcodes were generated on the basis of their structural subtypes from a statistical investigation of their *δ*_H_ and *δ*_C_ data in the literature. These reference barcodes allowed *in silico* identification of known triterpenes in enriched fractions obtained from an extract of *A. racemosa*. After dereplication, a differential analysis of heteronuclear single-quantum correlation (HSQC) spectra even allowed for the discovery of a new triterpene. The 2D barcoding concept allowed for the rapid dereplication of known compounds as well as the search for structural novelty.

#### 5.1.3 HMBC networking

Hubert *et al.* developed an elegant strategy featuring the networking theory for the exploitation of heteronuclear 2D NMR data in the context of natural crude extracts analysis.^84^ This *in silico* method leverages HMBC and HSQC spectra to extract short-range and long-range H–C correlations occurring in a carbon skeleton.

Briefly, an algorithm based on the community detection recovers individualized HMBC fingerprints from the HMBC data of a complex mixture. Then, the collected H–C correlations are represented as a network of NMR peaks. After the generation of clusters from the obtained NMR peak network, molecular structures are assigned by means of an in-house theoretical HMBC and HSQC correlation database. Although this strategy has been exemplified for the identification of entire molecules, one can imagine the application of this technique to link the HMBC networking spectral features to substructures following a similar strategy but now linking the clusters to partial structures for example based on highly interlinked proton and carbon atoms.

#### 5.1.4 Backbone topology determination

The Brüschweiler lab developed an elegant approach^85^ based on a combination of a ^13^C 2D-NMR technique (long time mixing time ^13^C CT-TOCSY) and a deconvolution algorithm (DeCoDeC^86^) that will identify traces that are unique for individual mixture components. Interestingly, the carbon connectivity information will be reconstructed from the assembly of each consensus trace using short mixing time CT-TOCSY and COSY.^87^ This strategy was applied to the characterization of the metabolites of ^13^C-enriched lysate of *E. coli* cells leading to determine their carbon backbone topologies coined as “the topolome”. The latter was dominated by carbon topologies of carbohydrates (34.8%) and amino acids (45.5%) that can act as a foundation to assemble more complex metabolites.

#### 5.1.5 SMART

^1^H–^13^C heteronuclear single quantum correlation (HSQC) spectra, which provide correlations between a carbon and its attached protons, are key data for structural elucidation of chemical compounds. Small molecule accurate recognition technology (SMART), of which the prototype was introduced in 2017,^88^ is a deep convolutional neural network (CNN)-based tool for automated annotation of compounds from HSQC spectra. SMART takes a HSQC spectrum as an input and gives a list of estimated compound structures based on a (deep) CNN model trained with multiple HSQC spectra (2054 for the prototype and 53 076 for version 2.0) of previously reported natural molecules. In 2020, the first application of SMART 2.0 for a mixture analysis was reported.^89^ In this work, SMART successfully estimated that the active fraction of *Symploca* sp. extract would contain a macrolide compound similar to swinholide; and the major swinholide class molecule, symplocolide A, was subsequently isolated and structurally determined from this fraction.

### 5.2 Linking spectral features to bioactive substructure fingerprint: pharmacophoric deconvolution

A continual quest of bioactivity-guided natural product discovery workflows lies in the development of key methods for connecting small molecule structures with their biological functions. In this regard, an interesting approach based on statistical HeteroCovariance Analysis (HetCA) has been introduced.^90^ Furthermore, several studies have shown that differential analyses of 2D NMR spectra (DANS) of natural product extracts can be highly effective for associating small molecules with specific biological properties.^91^ It is worth noting that, as is true for all unsupervised methods developed for MS and NMR, the exploitation of the NMR spectral data still relies on manual interpretation with human expert knowledge.

#### 5.2.1 ELINA: bioactivity correlation of NMR signals

Grienke *et al.* developed ELINA (Eliciting Nature's Activities), a strategy based on statistical heterocovariance analysis (HetCA) of ^1^H NMR spectra detecting spectral features that are positively (“hot”) or negatively (“cold”) correlated with bioactivity prior to any isolation.^92^ ELINA is demonstrated with the discovery of steroid sulfatase inhibiting lanostane triterpenes from a complex extract of the polypore fungus *Fomitopsis pinicola*. As a way to extract the spectroscopic signals related to the “hot” features, STOCSY analysis was performed to generate the fingerprint of the active component in the mixture. Indeed, the ELINA approach efficiently extracts the spectral fingerprint of the bioactive component from the data.

#### 5.2.2 Plasmodesma: automatised pharmacophoric deconvolution

To face the avalanche of data that derives from the manual comparison of metabolomics studies or natural extracts screening, Delsuc *et al.* developed a computer program, nick-named Plasmodesma^93^ allowing the autonomous, unsupervised processing of a large corpus of 1D and 2D NMR spectra acquired in different conditions. The capabilities of this tool were extended to be able to extract the spectral fingerprint of a molecule of interest from a set of NMR experiments through a simple linear regression, leading to pharmacophoric deconvolution.^94^ Briefly, this tool (available at: https://plasmodesma.igbmc.science) handles the NMR data as statistical entities, and uses curated bucket lists rather than peak lists for detecting signal variations which correlate with the activity. Next, the results are displayed in an interactive visualization manner in which an NMR spectroscopist should be able to easily recognize molecular patterns.

### 5.3 Linking spectral features to unusual substructures

#### 5.3.1 MADByTE

MADByTE, which stands for Metabolomics And Dereplication By Two-dimensional Experiments, is a computational tool for comparative analysis of NMR spectra from large sample sets.^95^ It uses data acquired by two different NMR experiments: ^1^H–^13^C HSQC and ^1^H–^1^H total correlation spectroscopy (TOCSY). TOCSY can provide information on spin system features, which is related to specific substructures of molecules; and MADByTE constructs an association network between spin system features and samples, which can be pure compounds, fractions, or extracts. From this, users can distinguish shared spin systems between samples and use the information for dereplication and recognition of unusual spin systems that are likely to belong to yet unseen chemical substructures.

#### 5.3.2 Atomic novelty scoring

Another ^1^H–^13^C HSQC experiment-based method was suggested by Duggan *et al.* in 2019.^96^ The authors established a ^1^H–^13^C HSQC database using publicly available spectra in the Human Metabolome Database (HMDB)^97^ and the BioMagResBank.^98^ As a result, the authors enlisted 10 308 ^1^H–^13^C HSQC peaks from 1207 spectra. These peaks were supposed to be common or usual peaks in general molecules. Then, the authors calculated Euclidean distances between all peaks in the profiled spectrum (of single compound or crude extract) to the closest peak in the database. Since the ^1^H or ^13^C chemical shifts represent the chemical environment of each atom in a molecule, this method can estimate the novelty of each atom of molecules; thus, this method can be used to prioritize a sample of interest or a target compound, which is expected to contain a novel substructure.

## 6 Toward NMR-based compound class prediction through CASE

In mass spectrometry approaches, recent computational advances have created several tools that allow to annotate metabolomics profiles with substructures (Section 2) and chemical classes (Section 3). With the amazing improvements in molecular structure recognition using NMR prediction tools, fine determination of metabolite structure is enabled using the modern approach of numerical chemistry. Recent advances in software packages that are used in computer assisted structure elucidation (CASE) have minimized the prediction of ^1^H and ^13^C signals to a precision of 1.2 ppm mol RMSE for ^13^C and 0.4 ppm mol RMSE for ^1^H shifts.^99^ This allows for NMR-based metabolomics computational tools to start recognizing substructures in complex metabolite mixtures, especially from 2D-NMR experiments (Fig. 4). This raises the question whether NMR-based chemical compound class predictions will be possible as well. So far, no dedicated and comprehensive tool to do this akin to MolNetEnhancer or CANOPUS for mass spectrometry data has been proposed, but some early and recent examples of successful approaches for ^13^C-based automated chemical compound classification were introduced, *i.e.*, CARAMEL,^75^ SENECA,^100^ and an approach based on a XGBoost classifier,^101^ as well as functional group recognition.^102^ For example, the XGBoost classifier performed well in the automated recognition of nine natural product classes yielding performances above 80% accuracy for most classes on test data: sesquiterpenoids, triterpenoids, and flavonoids could be distinguished well. Furthermore, the presence of a glycoside moiety could be accurately predicted as well. We do note that ^13^C-NMR chemical shifts are indeed well predicted using quantum mechanics (Hartree–Fock [HF] or density functional theory [DFT]) or machine learning (hierarchical organization of spherical environments [HOSE]).^103^ The (further) automation and extension of the above-mentioned workflows, in particular toward the use of 2D-NMR spectra, is hampered by the challenging process of 2D-NMR peak picking: existing methods are indeed based on the intensities or the area of the NMR peaks and often fail in identifying peaks with low intensity or overlapping ones.^104^ Here, we expect that the development of machine learning enhanced spectral feature recognition, for example based on computer vision (SCRV),^105^ may play an important role in automated ^1^H and ^13^C NMR data extraction from experimental data, but also from NMR data presented in literature to further expand spectral databases.

Next to improved spectral feature recognition that will boost automated workflows, we recognize a number of steps that the field could take and work on to further this area. Firstly, the extension of NMR spectral libraries such as NMRShiftDB^106^ will help to map NMR signals to structures and their substructures and molecular fingerprints. The latter two can be used for automated chemical compound classification using ontologies such as ChemOnt (from ClassyFire) or NPClassifier. Such extensions could also be done *in silico*^107^ where the predictions are based on machine learning and incorporate an uncertainty measurement. When incorporated into NMR databases, data mining strategies could be used to link experimental and confidently predicted NMR shift signals and patterns to chemical compound classes. This could also result in augmented spectral databases and recently the PNMRNP database derived from UNPD was introduced to the community of natural product chemists.^108^ In a similar spirit, the chemical shift prediction and matching capabilities of NMRfilter at the structural level could be extended to the substructure level.^109^ A related KnapsackSearch was also introduced as a database generator that provides taxonomically focused libraries of metabolites to narrow down the search space in a “bio-logical” manner. The importance of taxonomy in the identification of compound class was further highlighted in this recent contribution^110^ by Rutz *et al.* where the authors present an open natural products database LOTUS (naturaL prOducTs occUrrences databaSe) in which the biological occurrences of over 500 000 natural product metabolites collected from various sources^111,112^ are now extensively documented.

## 7 Other analytical methods

Although NMR and MS are two major workhorses for mixture analysis these days, other spectroscopic methods are also occasionally used for class or substructural annotation of metabolites. For example, using a photodiode array detector (PDA), ultraviolet and visible wavelength (UV-VIS) absorption spectra can be obtained that have been used for rough class annotation of chromophores for decades, and are still used for identification of specific molecular classes, or identification of metabolites in specific taxa; *e.g.* flavonoids in plants^113^ or phenolic compounds in lichens.^114^ This knowledge is generally applied by manual inspection, but a recent study on flavonoid analysis demonstrated that UV-VIS-based annotations also can be computionalized.^115^ Vibrational spectroscopic methods, such as near infrared (NIR), Fourier transformed infrared (FTIR) and Raman spectroscopy, have their own advantages; they can provide rapid, high-throughput, and non-destructive analysis. Vibrational spectroscopic methods provide highly overlapped signals from multiple molecules, so they are typically applied as “fingerprint” methods rather than to structurally identify individual constituents. However, if they provide fundamental information about the presence of functional groups in molecules, they have enough potential to be applied to substructural identification in the metabolomics workflow, especially of active groups that are often crucial for bioactive or toxic properties of a molecule. Recently, the combined input of FTIR and MS spectra was used to train a deep learning model to recognize the presence of functional groups such as carboxylic acid, aromaticity, and the ester group.^116^ The authors concluded that FTIR spectra could in many cases reliably annotate such functional groups, but MS did offer additional information in a fair number of cases. In another recent example, infrared ion spectroscopy linked to mass spectrometry could readily separate enantiomeric *N*-acetylhexosamines identified in body fluid samples.^117,118^ As typical natural mixtures contain numerous isomeric molecules, infrared ion spectroscopy is expected to be of great added value for molecular identification.

## 8 The future of computational metabolomics in natural products discovery

### 8.1 Toward a database of annotated structural motifs

The various examples provided here make clear that over the last two decades enormous steps have been taken in metabolomics analyses to go from file-by-file analysis toward integrated multi-file analyses of entire experiments. Several parts of the metabolomics analysis workflow have been automated effectively such as preprocessing and noise filtering. However, metabolite feature recognition and metabolite annotation and identification remain bottlenecks as they still require substantial manual intervention and expertise. We argue here that machine learning approaches and network and substructure-based approaches are key strategies to come to the higher level of automated analysis needed to enable larger-scale metabolomics analysis compatible with the information-dense spectral data that current and the future instrumental advances will bring. Machine learning approaches could be used to more accurately detect metabolite features in mass spectrometry and especially NMR data. The automated recognition of signals in 2D-NMR spectra is far from a solved challenge and with the increasingly available annotated datasets that will provide sufficient training and test material, the manual process of signal recognition could be automated. It is interesting to note that there is currently no central server to upload NMR spectra, and process, analyze, and visualize NMR results supported by a public spectral library,^119^ whilst several of such platforms have emerged for mass spectrometry-based metabolomics data.^21,120,121^ We do note that initiatives like nmrML^122^ and NMReDATA^123^ have been proposed which we believe will in turn spark the development of computational NMR-based metabolomics tools.

**Fig. 5:**
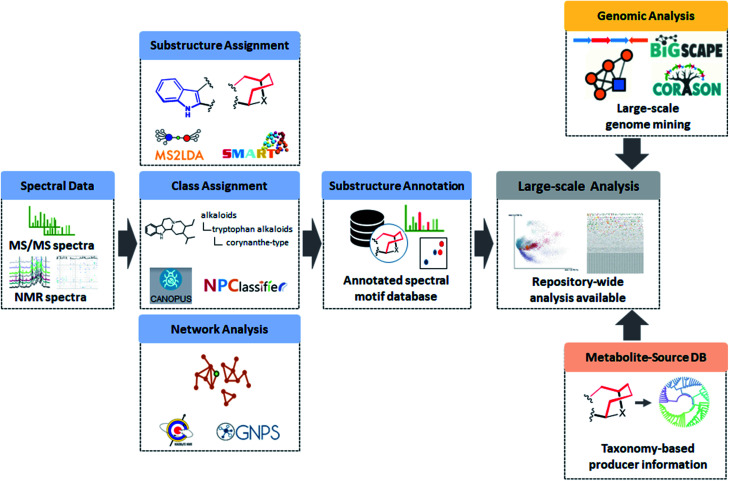
Technical advances on both the MS and NMR side both result in unprecedented chemical insights in complex metabolite mixtures. The recent surge in available computational metabolomics tools to extract substructure and chemical class information and to build networks based on spectral data is remarkable. Looking into the nearby future, we foresee an increased demand to perform large-scale repository-wide metabolomics analyses and the establishment of a database focused on substructure motifs that can be recognized in MS (MS/MS) and NMR (2D-NMR) data will be a key pillar to do so to annotate all the key building blocks in complex mixtures that form the bioactive components. Here, we recognize that the MS field has started to organize itself over the last years with centralized repositories and analysis workflows for processing, comparison, and visualization, whereas in the NMR field this is still largely absent, although there are some encouraging signs based on recent literature. Furthermore, this will also highlight the opportunities for crosstalk between those two analytical routes. We note that both chemical class assignments and network analysis can be used on their own to perform large-scale analyses as well. We also note how complementary information, for example from genomics analyses where genome mining tools are predicting structural features with increasing accuracy, will further help in structural annotation of complex chemical mixtures. This will work especially well in tandem with well-curated taxonomic resources, a field in which we observe increasing efforts lately. This will not only provide organism-tailored candidate metabolite lists for omics experiments, but also complement the information from genome mining and allow researchers to further fill in the gaps in the specialized metabolomes of organisms. Ultimately, we believe that the here described tools and our perspective on their future developments will altogether transform metabolomics datasets from collections of spectral features to biochemical interpretable representations.

In mass spectrometry, the better discrimination between real metabolite features and artefact signals remains a topic of much attention and debate, with ion identity networking as one of the latest approaches to declutter the complex and dense mass spectral datasets.^124^ After establishing a good peak list, the challenging structural annotation step begins. Here, the more efficient mining for unique substructure motifs will facilitate (i) the prioritization of novel chemistry in complex metabolite mixtures, and (ii) the structural elucidation procedure as such substructure motifs can often be related to biosynthetic or chemical building blocks of the metabolites. In mass spectrometry, tools like MS2LDA^16^ and MESSAR^23^ have started to recognize substructure motifs based on spectral data, and MotifDB^20^ is able to store annotated substructure motifs facilitating their reuse for future structural annotation purposes; however, in many cases, the structural annotation and verification of the substructure chemistry still relies on analytical experts that need to identify the structural motifs by linking them to elemental formulas and structures or chemical compound classes. In NMR, 2D-NMR spectra have started to be exploited to recognize substructure motifs,^125^ but here the automated recognition of NMR signals may hamper progress in this area. Looking into the future, we can envision the implementation of a database of MS and NMR annotated substructure motifs that can be used to populate the majority of metabolite features in your experiments with structural information supporting larger-scale metabolomics analyses (Fig. 5).

### 8.2 The integration of LC-MS/MS and 2D-NMR approaches

The remarkable increase in available computational metabolomics workflows has boosted many scientific disciplines that rely on small molecule measurements; however, it has also come with new challenges. In order to combine the analysis of mass spectral and NMR-based experiments, the output of various tools needs to be combined. This is far from trivial, even within these two analytical fields, as was demonstrated by the development of MolNetEnhancer^36^ that brings together the output of various metabolome mining and annotation tools, thereby also facilitating the structural annotation of substructure motifs. We note that the various input and output formats that exist can hamper integrated analyses with the risk of creating “analysis silos” that on itself are great data analysis ecosystems but their output is hard to effectively combine with other tools. A possible solution is to make such tools compatible with the various ecosystems; however, this puts a substantial additional burden on the tool developers.

Existing approaches that combine LC-MS/MS and 2D-NMR analysis typically perform the analytical analyses separately for each platform and then combine that knowledge manually during the structural elucidation process,^126^ or select candidates based on one method and then further reduce that list using the other method,^127^ for example by annotating relevant substructures from LC-MS/MS data or MSn data^128^ and look for them in the NMR data.^125^ To perform simultaneously integrated LC-MS/MS and 2D-NMR analyses, the initial – very different – spectral data needs to be converted into a list of metabolite features with spectral and substructure properties that can be compared across and linked. We foresee a possible route by doing mixture analysis and applying (2D-)NMR and LC-MS/MS on the same samples and then combine and link the output of MS2LDA and MADByTe or the approach described by Kuhn *et al.*^125^ where prediction of 2D-NMR signals is used but then focused on the recognition of substructure motifs. Instead of first extracting substructure information from one method and then doing a targeted search to find them back in the other method, such an approach would enable increased crosstalk between MS and NMR through substructure linking. It has to be noted that the availability of the necessary equipment to perform both MS and NMR based analyses may hamper the uptake of such an integrated approach, as well as the costs to run and maintain such platforms or to outsource the measurements. Finally, to train algorithms in the effective linking of substructure data obtained from MS/MS and 2D-NMR, there is a need for the increased availability of well-curated datasets of complex mixtures with known constituents. Nevertheless, we foresee that such an approach can become very powerful in prioritizing novel chemistry and accelerating its structural elucidation, especially if supported by a database of annotated substructure motifs as discussed above.

### 8.3 Pathway and taxonomy supported metabolite annotation

Metabolites are seldomly present in isolation in complex mixtures, as they are typically linked to other metabolites through shared biochemical pathways or building blocks. To make effective use hereof, it is important to consider the biosynthetic capabilities of the organisms present in your experiment. For example, the concept of taxonomically informed scoring makes use of the presence and absence of metabolites across species and genera, thereby reranking candidate structure lists.^63^ The whole idea of chemotaxonomy arises from the necessity for a living organism to produce a set of compounds linked to its gene pool. We thus believe the recent development of the well-curated compound databases NP Atlas^129^ for microbial metabolites and LOTUS^110^ for (mainly) plant-based metabolites are essential for the well-functioning of such approaches. The concept of metabolite consistency^130^ then uses the knowledge of known biochemical pathways to assign the most likely candidate structure by eliminating theoretical options that do not fit with reported enzymatic activity. Pathway analysis is used to extract possible active pathways, often in combination with comparative metabolomics (see Section 8.4) based on known biochemical pathways or based on prediction of their presence. For example, NICEpath uses the concept of conserved atom ratio to detect plausible reaction pathways in large biochemical networks,^131^ and PALS decomposes known metabolic pathways that could be present in the samples or metabolite sets representing possible pathways by using metabolomics data from various conditions.^132^ An important limitation of the above strategies is the bias most of these approaches will have toward what we already know, as it is very challenging to acknowledge unexpected biochemical transformations through such strategies. In that sense, the concept of differentially expressed metabolite sets (as enabled by PALS) that contain metabolites grouped based on their spectral similarity is an interesting exception; however, such metabolite sets do not necessarily represent biochemical pathways and further validation steps are thus required.

### 8.4 Comparative metabolomics and metabolite annotation

Comparative metabolomics typically aims to detect differences in metabolite profiles between samples or differential conditions. Essential parts of such a workflow are the peak picking of MS or NMR metabolite features in all samples and the alignment of the same metabolite features across all samples to enable the statistical comparison based on peak heights or areas of metabolite features. Following such an approach, metabolite features can be linked to certain phenotypes, activities, or functions, and thus prioritized for further analyses, often including their structural elucidation. Recently, the concept of comparative mass spectrometry-based metabolomics was seamlessly integrated with molecular networking resulting in Feature-Based Molecular Networking (FBMN).^133^ Following this analysis workflow enables the more accurate detection of isomers in molecular networks, and the use of reliable quantitative values for metabolite feature abundance. This in turn allows for interesting future applications such as the complementary use of correlation-based network edges in the molecular network that connect nodes that “behave similarly” across the sample types investigated. When combined with substructure-based workflows, a similar workflow could yield metabolite substructures that are enriched in particular sample types or correlated to a specific bioactivity based on their presence and absence across many phenotyped samples. Very recently, Qemistree was introduced that allows the comparison of MS/MS-based metabolite profiles in a chemically-informed manner.^43^ Ideally, when comparing metabolite profiles, one would like to acknowledge that samples that contain a high proportion of similar chemistry (*i.e.*, flavonoid glycosides), but not many completely identical components, are still more related than samples that contain different chemistry altogether (*i.e.*, flavonoids *versus* terpenes). To achieve this, molecular structure fingerprints were first predicted from the MS/MS spectra using SIRIUS-CSI:FingerID,^27^ followed by their comparison through hierarchical analysis that results in a tree akin to when analyzing and visualizing the relatedness of DNA sequences. Here, metabolite features with similar molecular fingerprints end up close together in the phenetic tree. When performed on a metabolomics experiment and decorated with sample metadata and chemical ontologies, this allows to gain insights in sample relatedness through the relatedness of their individual constituents. Similar approaches for NMR-based metabolomics experiments are expected to provide similar advantages, especially when the computational workflows that support large-scale analyses will further mature.

### 8.5 Structural diversity and the limitations of spectrum-based analysis

Whilst many powerful tools and examples were provided in this review, spectrum-based analyses have their limitations. In mass spectrometry, the MS/MS fragmentation that many tools now heavily rely on for structural information happens in the gas-phase in collision cells of mass spectrometers. As a matter of fact, and despite some successes,^25^ not much is understood from gas-phase chemistry and predicting when and how metabolites fragment remains very challenging, especially for electrospray ionization (ESI)-based collision induced dissociation (CID) types of fragmentation. In NMR, the complexity of 2D-NMR spectra with lots of overlap make it challenging to automate peak-picking and there is quite a sensitivity gap with mass spectrometry.

Furthermore, chemically divergent yet very relevant compound classes like alkaloids and terpenoids pose interesting questions in relation to network and substructure-based approaches: how to comprehend these chemical compound classes from a substructure/chemical class point of view? Historically, the different biosynthesis routes have determined various subclasses of alkaloids and terpenoids, but these are not always easily recognized by spectral features as sometimes a tiny structural difference is hardly visible in the analytical data but needs a substantially different enzymatic route, or *vice versa*: a tiny structural difference due to the decoration of a scaffold causes drastic changes in the spectra.^128^ Such situations are difficult to resolve and may signpost the border area of where spectral-based analyses are useful. However, with the increase of publicly available data and the development of novel tools such as the new mass spectral similarity measure Spec2Vec as well as alternative networking-based approaches,^45,134,135^ it could well be possible to group together structurally similar metabolites (according to historical reasons and/or biosynthetic routes) taking into account that mass spectral features that are not exactly similar could still be related to each other. Moreover, with an increased number of annotated datasets, supervised machine learning approaches could further improve on the current performance of Spec2Vec.

### 8.6 Breaking barriers

Another route to improve on metabolite annotation performance is to gain complementary information about the same samples from other sources such as genomics. In natural product discovery, genome mining tools such as antiSMASH^136^ can mine genome and metagenome sequences for their biosynthesis potential and return predicted biosynthesis gene clusters that likely encode for the production of specialized metabolites. Tools like BiG-SCAPE^137^ and BiG-SLICE^6^ can, at a respectively smaller and larger scale, group those biosynthesis gene clusters in gene cluster families that are likely to produce structurally related metabolites. One can then envision that such gene cluster families could be linked to molecular families obtained from metabolomics data,^138^ for example through pattern-based genome mining.^139^ That would help to find the likely producers of specialized metabolites in complex samples, as well as to gain complementary structural information such as about the stereochemistry of chiral centers. In recent years, the first strain correlation and feature-based scores have been developed,^15^ as well as novel scores,^140^*i.e.* as proposed by Soldatou *et al.*,^141^ to charter chemical diversity in polar bacteria. Such attempts are largery enabled by cataloguing (i) known and validated biosynthesis gene clusters (BGCs) encoding for the production of specialized metabolites in MIBiG,^142^ (ii) known and curated metabolites with metadata on their origins in databases such as NP Atlas,^129^ and reference spectra in open libraries such as those from MassBank^143^ and GNPS,^21^ and will be further driven by their integration and initiatives such as the paired omics data platform that brings together sequenced genomes and metagenomes with LC-MS/MS metabolomics profiles that are available in the public domain.^144^ For example, the NPLinker framework has already adopted a workflow to directly read in data from the paired omics data platform to then perform various scores to rank and prioritize BGC-MS/MS spectral links for further study.^140^ Looking into the future, substructure-based workflows could further enhance such rankings by linking the substructure annotations of MS2LDA and MESSAR with those from the antiSMASH subclusterblast and by statistical recognition of genes that could encode for the production of a substructure.^145^ Alternatively, the predicted antiSMASH compound classes (non-ribosomal peptides, polyketides, *etc.*) could be used to prioritize MS or NMR spectra that are predicted to be of those compound classes through the use of MolNetEnhancer or CANOPUS or NMR-based alternatives.

Most of the attention in this review was spent on the profiling of non-volatile specialized metabolites. However, nature produces many volatiles and recently an open platform for GC-MS data analysis and library matching was established.^54^ Analogous to the above-described options to link MS/MS-based substructures with those observed in 2D-NMR, we could envision something similar for GC-MS to develop.

The structural annotation of metabolites is usually only the start: it is typically the functional annotation that is most relevant to the biological or biochemical research question. Well curated and annotated datasets are crucial to make large-scale metabolomics analyses effective in functional analysis. In that respect, the launch of ReDU in 2020 symbolizes the beginning of repository-level analysis of MS/MS datasets.^146^ ReDU allows the reanalysis of public MS/MS datasets with metadata using a controlled vocabulary, which enables researchers to project their data on all the available public data in ReDU and assess its chemical uniqueness. The other way around, researchers can also select a specific subset of the LC-MS/MS data, for example all fungal datasets, when they want to study fungal chemical diversity. The next step is to do the same thing for MS/MS spectra: when metadata at the spectral level is consistently collected and stored, this will allow researchers to better track the origin and possible functions of the metabolites in their profiles. Moreover, through network and substructure-based approaches, such spectral metadata can also be propagated to related metabolites in the same dataset. It is encouraging to observe that also on the NMR side there is an increased attention for sample metadata.^147^

With analyses happening at increasingly larger scales, in-depth repository-scale analyses are becoming within reach. To visualize such amounts of data and efficiently extract the relevant information from it, novel algorithms are needed that are scalable, *i.e.*, in clustering all MS/MS spectra, that speed up analysis time, and that can visualize the resulting data structures to enable analysis and biochemical interpretations. Such developments that lead toward repository-scale analyses will in turn increase the benefits of sharing well-documented datasets in the public domain, thus changing data sharing from a perceived (time) burden into an actual benefit.

We do note that the growth in the number of available computational tools and the increasing density and size (number of data files) of typical metabolomics experiments can come with high computational costs. Whilst numerous tools can be run on a fairly normal laptop or desktop computer, there are several tools that benefit from dedicated servers with sufficient memory and cores available. Furthermore, many tools have not been fully optimized for performance; we refer to the review by Chang *et al.* for an extensive overview of these and other opportunities and limitations of current metabolomics tools.^148^ It is encouraging to see that platforms such as GNPS^21^ put effort in integrating novel workflows (many of which highlighted in this review) into their workflows. This not only makes them easier to integrate in metabolomics analysis, but also democratizes their use as the users can also access dedicated analysis servers linked to GNPS that they otherwise may not have had access to. We expect that with increased popularity of the described approaches, more of such analysis services are likely to appear.

## 9 Conclusions & final perspectives

Computational metabolomics approaches have started to change the metabolomics field by automating various aspects of typical metabolomics workflows and thereby enabling large-scale metabolomics analysis. Together with the current increase in publicly available datasets, this has also shifted the focus of many studies towards unknown metabolites that are not fully described and catalogued in databases yet. In this paper, we show how substructure and network-based metabolomics approaches can cause a paradigm shift in the annotation level of these yet unknown metabolites in the forthcoming years by leveraging structural, chemical compound class, and substructural information from MS/MS and NMR spectral data. Once structural motif databases are sufficiently populated, they in turn will spark the development of new tools to accelerate the elucidation of entire structures and metabolic pathways based on the available spectral data in various conditions. We expect that the linking of MS/MS spectra to information obtained from genome mining positively contributes to the annotation power of metabolomics data. We foresee that the impact of machine learning-based approaches will further increase with the increased availability of metabolomics data that can serve as training and test data to improve the performance of spectral library matching and to create biochemically interpretable mass spectral networks. Together with well-curated and consistent reported metadata, this will open up new avenues to directly link taxonomic and functional annotations to spectral data. Altogether, we conclude that networking and substructure-based computational metabolomics analysis workflows have already started to form an essential part of the future of metabolomics in which large-scale metabolomics datasets can be rapidly transformed into present and active metabolic pathways and metabolite groups with annotated functions – a necessity to efficiently apply wide-screen metabolomics approaches in large-scale natural product discovery studies and other scientific disciplines.

## 10 Author contributions

Mehdi A. Beniddir: conceptualization, methodology, data curation, writing – original draft, review & editing, validation, visualization. Kyo Bin Kang: conceptualization, writing – original draft, review & editing, visualization. Grégory Genta-Jouve: data curation, writing – original draft, review & editing, validation, visualization, Florian Huber: methodology, writing – original draft, review & editing, Simon Rogers: writing – original draft, review & editing, Justin J. J. van der Hooft: conceptualization, writing – original draft, review & editing, supervision, validation.

## 11 Conflicts of interest

There are no conflicts of interest to declare.

## Supplementary Material
